# Characterization and Comparative Analysis of the Complete Chloroplast Genome of the Critically Endangered Species* Streptocarpus teitensis* (Gesneriaceae)

**DOI:** 10.1155/2018/1507847

**Published:** 2018-03-25

**Authors:** Cornelius M. Kyalo, Andrew W. Gichira, Zhi-Zhong Li, Josphat K. Saina, Itambo Malombe, Guang-Wan Hu, Qing-Feng Wang

**Affiliations:** ^1^Key Laboratory of Aquatic Botany and Watershed Ecology, Wuhan Botanical Garden, Chinese Academy of Sciences, Wuhan 430074, China; ^2^University of Chinese Academy of Sciences, Beijing 100049, China; ^3^Sino-Africa Joint Research Center, Chinese Academy of Sciences, Wuhan 430074, China; ^4^East African Herbarium, National Museums of Kenya, P.O. Box 45166-00100, Nairobi, Kenya

## Abstract

*Streptocarpus teitensis* (Gesneriaceae) is an endemic species listed as critically endangered in the International Union for Conservation of Nature (IUCN) red list of threatened species. However, the sequence and genome information of this species remains to be limited. In this article, we present the complete chloroplast genome structure of* Streptocarpus teitensis* and its evolution inferred through comparative studies with other related species.* S. teitensis* displayed a chloroplast genome size of 153,207 bp, sheltering a pair of inverted repeats (IR) of 25,402 bp each split by small and large single-copy (SSC and LSC) regions of 18,300 and 84,103 bp, respectively. The chloroplast genome was observed to contain 116 unique genes, of which 80 are protein-coding, 32 are transfer RNAs, and four are ribosomal RNAs. In addition, a total of 196 SSR markers were detected in the chloroplast genome of* Streptocarpus teitensis* with mononucleotides (57.1%) being the majority, followed by trinucleotides (33.2%) and dinucleotides and tetranucleotides (both 4.1%), and pentanucleotides being the least (1.5%). Genome alignment indicated that this genome was comparable to other sequenced members of order Lamiales. The phylogenetic analysis suggested that* Streptocarpus teitensis* is closely related to* Lysionotus pauciflorus* and* Dorcoceras hygrometricum*.

## 1. Introduction

*Streptocarpus* Lindley (Cape primroses) are herbaceous plant species of the family Gesneriaceae and exhibit either annual or perennial lifeforms. The genus hosts approximately 176 species [1], distributed in Madagascar, Comoros islands, and some regions of Africa [2, 3], with the regions sharing no single species [4]. The genus, like most members of the family Gesneriaceae, hosts some species of great horticultural importance such as* Streptocarpus ionanthus* which contribute a large percentage to the world's flower industry [5].

*Streptocarpus teitensis* [6], previously known as* Saintpaulia teitensis*, is an endemic of Taita hills, Kenya, the northernmost stretch of the Eastern arc mountain forests. The species has experienced a drastic reduction in the distribution range over the past, and it was recorded to be restricted to a single site (<2.5 km^2^) in the wild, Mbololo Hill [7], although more than half of the range has deteriorated of late (personal observation, 2017). This restriction in distribution coupled with other threats facing this species has led to its critically endangered conservation status under the IUCN red list (http://www.iucnredlist.org/). Additionally, none of the species in this genus has been sequenced of its complete chloroplast genome. It is therefore essential to obtain the complete chloroplast genome sequence as a step towards generating genomic resources that can aid future phylogenomic studies within this family.

The chloroplast is crucial for plant cell processes such as photosynthesis [8–10] and physiological and development processes such as leaf and root development [11–13]. This organelle owns a single circular DNA, which in angiosperms is four-structured made of two duplicates of inverted repeat (IR) regions, one large single-copy (LSC) region, and one small single-copy (SSC) region [14]. Almost all chloroplast (cp) genomes studied show a relatively limited size range occurring between 120 and 160 kilobase pairs [10, 14–16] and comprise 110–130 genes, of which four are ribosomal RNA genes, ~80 are protein-coding, and ~30 are transfer RNAs [17].

Complete sequenced chloroplast genomes have numerically improved of late owing to the technical developments in DNA sequencing [11, 18] such as the Next-Generation Sequencing [19]. The availability of chloroplast genomes enables researchers to understand the evolution of the genomes, the structural organization, genes present, gene order, and the nucleotide alignment [18]. It has also made noteworthy contributions to phylogenetic studies of a significant number of plant families and determined their evolutionary links [11]. This has been made possible by the fact that chloroplast genome has a simple and stable genetic structure [20]. Over 1000 cp genomes are already completely sequenced and explained (https://blast.ncbi.nlm.nih.gov/) since 1986 when the first chloroplast genome was sequenced in Tobacco [10]. However, since there are only three sequenced members of the family Gesneriaceae, identifying the unique features of the family's chloroplast genome can lead to deceptive estimates of species relations [21]. Thus, complete chloroplast genomes from additional members of the family are essential for comparative analysis to show structural variations among the genomes. The aims of this study were to present the first whole chloroplast genome sequence of the genus* Streptocarpus* and to conduct comparative analyses against close relatives.

## 2. Materials and Methods

### 2.1. Plant Material, DNA Extraction, Sequencing, and Assembly

Leaf samples were obtained from* S. teitensis* in Taita hills, Kenya, and immediately dried in silica gel [22] to preserve until DNA extraction. Voucher herbarium specimens (Voucher Number: SAJIT_006426) were deposited at the East Africa Herbarium (NMK) and Herbarium of Wuhan Botanical Garden (HIB) for future reference purposes. Total genomic DNA of one individual was extracted from 100 mg of leaves via the MagicMag Genomic DNA Micro Kit (Sangon Biotech Co., Shanghai, China) guided by the manufacturer's instructions, after which the quality was assessed by electrophoresis on a 1% agarose gel. The complete genomic DNA was sequenced by the Illumina Hiseq 2000 Platform (Illumina, San Diego, CA), yielding 5,906,885,400 raw bases of which 5,883,619,800 were clean bases. In addition, the raw data was recognized through BLAST (https://blast.ncbi.nlm.nih.gov/) search against* Lysionotus pauciflorus* (GenBank Number: KX752081) sequences since it showed the highest similarity. This produced sequence contigs which were organized and plotted against the already published plastid sequences of* Lysionotus pauciflorus* also via BLAST (https://blast.ncbi.nlm.nih.gov/) with default settings, in order to acquire the chloroplast genome reads. These reads were then assembled into contigs in Velvet 1.2.10 [23]. An alignment of the resulting contigs with closest chloroplast genomes of* Lysionotus pauciflorus* and* Haberlea rhodopensis* (GenBank Number: KX657870) was done via BLAST (https://blast.ncbi.nlm.nih.gov/).

### 2.2. Genome Annotation

The assembled cp genome was annotated using the online program Dual Organellar GenoMe Annotator (DOGMA) [17], combined with manual alterations for the doubtful start and stop codons based on comparison with homologous genes from other sequenced chloroplast genomes. The annotation of the tRNA genes was verified using tRNAscan-SE [24]. The circular cp genome map was constructed by the use of online OGDRAW program [25]. Finally, the annotated genome sequences were submitted to the NCBI GenBank under Accession Number MF596485.

### 2.3. Genome Comparison and Phylogenetic Analysis

The genome features such as GC contents and size of* S. teitensis* cp genome were compared with the available three chloroplast genomes in Gesneriaceae (Table 5) and 11 other species representing nine different families within the order Lamiales (Table 6), to check for similarities and variations. Furthermore, all four species of Gesneriaceae (*Lysionotus pauciflorus*,* Haberlea rhodopensis*,* Dorcoceras hygrometricum* [1] (previously known as* Boea hygrometrica* as in [26]), and* Streptocarpus teitensis*) were used to do a comparative study on the expansion and contraction of the IR regions. This was achieved through analysis of the four junctions, a characteristic feature of angiosperm chloroplast genomes [27], using GenBank genome files.

Chloroplast genome sequence alignment was constructed using Mauve program [28] to check the gene order and sequence similarities/variations between 15 Lamiales cp genomes as in Tables 5 and 6.* Nicotiana tabacum* was added as reference genome (Figure 3) since it is considered to have the ideal angiosperm chloroplast genome structure. In order to determine the phylogenetic position of* Streptocarpus teitensis*, the cp genome annotation information of the species in Tables 5 and 6, together with two more Asterid species (*Sinadoxa corydalifolia* and* Coffea arabica*) included as outgroups, was obtained from the NCBI GenBank database. However, in this analysis the species* Cistanche deserticola* was not used since it has been recorded to have lost all the photosynthetic genes except* psbM.* A phylogenetic tree was constructed using Maximum Likelihood (ML) analysis executed in RAxML 8.0.20 following the instructions provided in the manual [29]. A total of 67 protein-coding genes common to all the selected chloroplast genomes were used. The jModelTest 2.1.7 program [30] was used to choose the finest appropriate substitution models GTR + I + G (p-inv = 0.47 and gamma shape = 0.93) based on the Akaike information criterion (AIC). A bootstrap study was achieved using 1000 replications.

### 2.4. SSR Analysis

Simple sequence repeats (SSRs) present in* Streptocarpus teitensis* cp genome were detected using MIcroSAtellite (MISA) identification tool (http://pgrc.ipk-gatersleben.de/misa/), by setting the number of repeats to eight repeat units for mononucleotide SSRs, five repeat units for dinucleotides, and three repeat units for trinucleotides, tetranucleotides, and pentanucleotide SSRs. A comparison was then done with the other three species of Gesneriaceae (Figure 2).

## 3. Results and Discussion

### 3.1. Gene Content and Organization of* Streptocarpus teitensis* Chloroplast Genome

**Figure 1:**
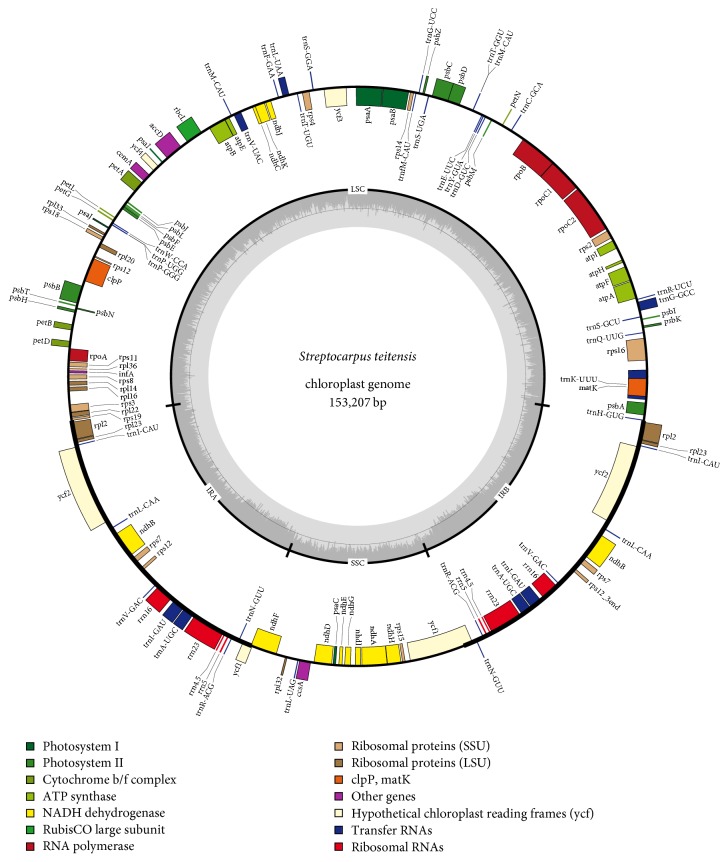
The gene map of the chloroplast genome of* Streptocarpus teitensis*. Genes drawn inside the map are transcribed clockwise, while genes drawn outside are transcribed counterclockwise. Different colors represent genes of different functional groups. Inverted repeats (IRA and IRB) are marked by the dark bold lines; GC and AT contents are, respectively, represented by the dark and light grey colors inside the map.

The whole chloroplast genome of* Streptocarpus teitensis* was found to be 153,207 bp in length, encompassing a large single-copy (LSC) region of 84,103 bp, a small single-copy (SSC) region of 18,300 bp, and a pair of inverted repeats (IRA and IRB) of 25,402 bp each which separate the two regions (Figure 1). Gene annotation revealed that the cp genome of* S. teitensis* contains 116 unique genes, of which 80 are protein-coding (69%), 32 are transfer RNAs (27.6%), and 4 are ribosomal RNAs (3.4%) (Table 1). This chloroplast genome maintained a constant overall GC content of 37.6% as observed previously in the family Gesneriaceae and also within the range of most completely sequenced chloroplast genomes of 30 to 40% [26]. Alike other dicot species,* S. teitensis* had the genes* rps19* and* trnH* at the points of IR/LSC junctions. A total of six protein-coding genes (*atpF*,* rps16*,* rpl2*,* rpoC1*,* ndhA*, and* ndhB*) and six tRNA genes (*trnA-UGC*,* trnK-UUU*,* trnG-GCC*,* trnI-GAU*,* trnL-UAA*, and* trnV-UAC*) had one intron each, while* clpP* and* ycf3* had two introns each (Table 2). The LSC region housed 62 protein-coding and 23 tRNA genes while the SSC region had 11 protein-coding and one tRNA gene. Additionally, 18 genes occurred as duplicates in the IR regions of which seven are protein-coding (*rpl2*,* rpl23*,* ycf2*,* ndhB*,* rps7*,* rps12*, and* ycf1*), seven tRNAs (*trnI-CAU*,* trnL-CAA*,* trnV-GAC*,* trnI-GAU*,* trnA-UGC*,* trnR-ACG*, and* trnN-GUU*), and the total four rRNAs. The GC content of IR regions (43.20%) is higher than that of LSC (35.54%) and SSC region (31.37%) (Table 3), a phenomenon which was observed by [31, 32] and explained to be brought about by the increased occurrence of GC nucleotides in the four rRNA genes. The* rps12* gene had the 5′_ end positioned in the LSC region and the replicated 3′_ end in the IR regions.

### 3.2. SSR Analysis

**Figure 2:**
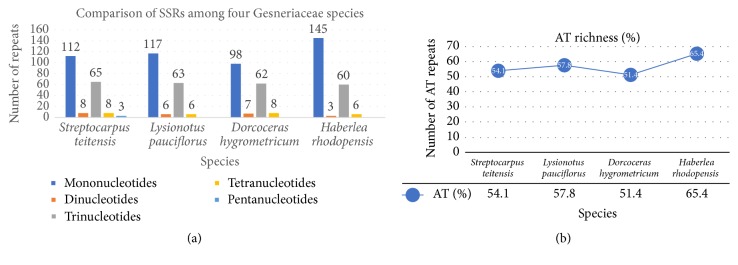
Comparison of SSR repeats (a) and AT repeats richness (b) among four Gesneriaceae species.

SSRs or microsatellites are tandemly repetitive DNA sequences that mostly measure <6 bp [33, 34], are spread all over the genome [32], and are categorized as mono-, di-, tri-, tetra-, penta-, and hexanucleotide [35]. Chloroplast SSRs (cpSSRs) are very polymorphic, reproducible, and plentiful in the genome [36] and are mainly useful in plant genetic studies [37]. A total of 196 cpSSR markers were detected in the chloroplast genome of* Streptocarpus teitensis*, with mononucleotides (57.1%) being the majority, followed by the trinucleotides (33.2%), dinucleotides and tetranucleotides (both 4.1%), and finally the pentanucleotides (1.5%) (Table 4). Compared to other Gesneriaceae species,* Haberlea rhodopensis* had the highest number of cpSSRs (214) while* Dorcoceras hygrometricum* had the lowest (175). In all the species, mononucleotide cpSSRs were the majority followed by the trinucleotides (Figure 2). There were no hexanucleotide repeats observed in the four studied Gesneriaceae species. Tetranucleotide repeats usually outdo the trinucleotides in number to some extent [32]. However, in this study, the trinucleotides (33.2%) were second to mononucleotides (57.1%). Kuang et al. [38] noted that short polyadenine (polyA or polyT) repeats are the major constituents of the simple sequence repeats occurring in the chloroplast genome, with tandem guanine (G) or cytosine (C) repeats being less frequent. This study had similar observations since, of the 112 mononucleotide repeats in* S. teitensis*, 106 were AT-type while only 6 were CG-type. The other three Gesneriaceae species exhibited this similarity since AT richness in each species was more than 50% of all the cpSSRs with* Haberlea rhodopensis* being the highest with 65.4%.

### 3.3. Comparative Chloroplast Genomic Analyses

This sequence represents the fourth complete chloroplast genome to be sequenced in Gesneriaceae. When compared to the firstly sequenced representatives,* Haberlea rhodopensis* [8],* Dorcoceras hygrometricum* [26], and* Lysionotus pauciflorus* [39], the four chloroplast genomes are comparable in terms of their gene content, genome organization, and structure despite some slight differences such as genome size (Table 5).* Lysionotus pauciflorus* (153,856 bp) was found to be the most extensive, followed by* Dorcoceras hygrometricum* (153,493 bp) and* Streptocarpus teitensis* (153,207 bp), while* Haberlea rhodopensis* (153,099 bp) was the shortest. It was also found that* Streptocarpus teitensis* had the shortest IR region, a phenomenon thought to be caused by the large size of the SSC region. Furthermore, comparison to other Lamiales placed the genome size of* Streptocarpus teitensis* between the largest and smallest of the genomes (Table 6), which were* Jasminum nudiflorum* (Oleaceae) with 165,121 bp and* Cistanche deserticola* (Orobanchaceae) with 102,657 bp, respectively. This inequality of the genome size can be explained, grounding the argument on the length of the LSC region as observed in earlier comparative studies on chloroplast genomes in Lamiales [32, 40].

The overall GC content between the four Gesneriaceae species was conserved (37.6–37.8%). In the SSC region,* Streptocarpus teitensis* and* Haberlea rhodopensis* had lower GC contents (31.4 and 31.7%, resp.). However, in the IRs the opposite was noted as* Lysionotus pauciflorus* and* Dorcoceras hygrometricum* had the lowest GC contents of ~40.7% compared to ~43.3% of* Streptocarpus teitensis* and* Haberlea rhodopensis.* Among Lamiales (Table 6),* Cistanche deserticola* had the lowest GC content (36.8%) while* Andrographis paniculata* and* Tanaecium tetragonolobum* had the highest (38.3%).

**Figure 3:**
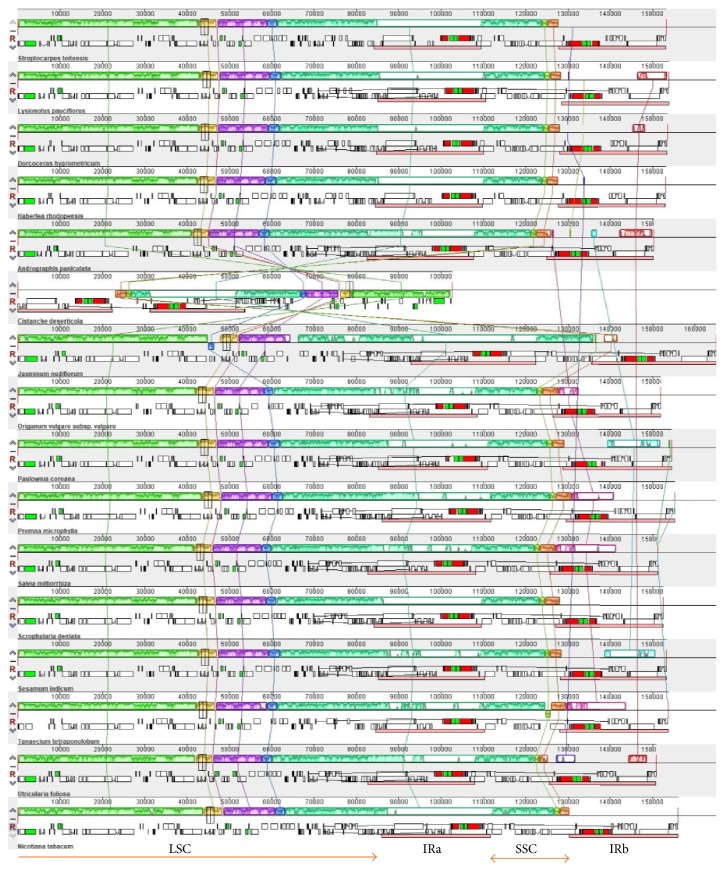
Mauve multiple alignment of 15 Lamiales, with* Nicotiana tabacum* set as the reference genome. Color-coded segments indicate regions that shared same genes across different species' genomes. The extent of sequence similarities is indicated by the colored parts inside each region. Lines connect regions with homologous sequences among two genomes.

The complete genome alignment using Mauve software was done between 15 species of Lamiales (Tables 5 and 6) and* Nicotiana tabacum* (Figure 3) which was added as reference genome since it is considered to have an ancestral arrangement of genes in angiosperms [41]. It was observed that many genome regions were conserved, with few variations in gene order, gene loss/gain events, and the direction in which the genes were transcribed. The inverted repeat regions (~90,000–110,000; ~130,000–160,000) showed the highest variations among the aligned chloroplast genomes, while the LSC (1–~80,000) and SSC (~110,000–130,000) were the most comparable. In addition, two species (*Cistanche deserticola* and* Jasminum nudiflorum*) had inversion events.* Cistanche deserticola* was the most notably different from other Lamiales, with all genes inversed and a reduction in genome size. The reduction in genome size was observed in a previous study and attributed to the loss of photosynthetic genes apart from* psbM* [42]. The inversion in* Jasminum nudiflorum* occurred on two genes* psaI* and* ycf4.* The four Gesneriaceae species in this study had a conserved gene arrangement and order and similar to other Lamiales as signified by the color blocks, with few variations in the sizes of the gene classes, drawing a conclusion of a close and conservative evolution of Gesneriaceae in the order Lamiales.

### 3.4. IR Expansion and Contraction

The IR regions have been observed to be potential distinguishing features among most angiosperms, as their expansions or contractions in or out of the single-copy regions are attributed to the different chloroplast genome sizes [43, 44]. The order in which genes are arranged in the junctions between the genome regions has also differentiated between species [45]. A comparable observation was made in photosynthetic orchids, whereby the chloroplast genomes exposed comparable structures but the IR and single-copy regions intersections together with the* ndh* genes displayed some variations [46]. Palmer and Thompson [47] discovered that chloroplast genomes which have lost the IR regions tend to be rearranged, findings which were duplicated by Strauss et al. [48], signifying that these regions function to uphold the structure of the genome. However, from both studies, it was not clear whether IR loss induced genome rearrangements and the latter study concluded that IR loss might reduce the resistance of the genome to rearrangements. A later study by Chumley et al. [49] found* Pelargonium x hortorum* genome to be reorganized despite containing the IR regions, further suggesting that IR loss does not induce genome rearrangements.

**Figure 4:**
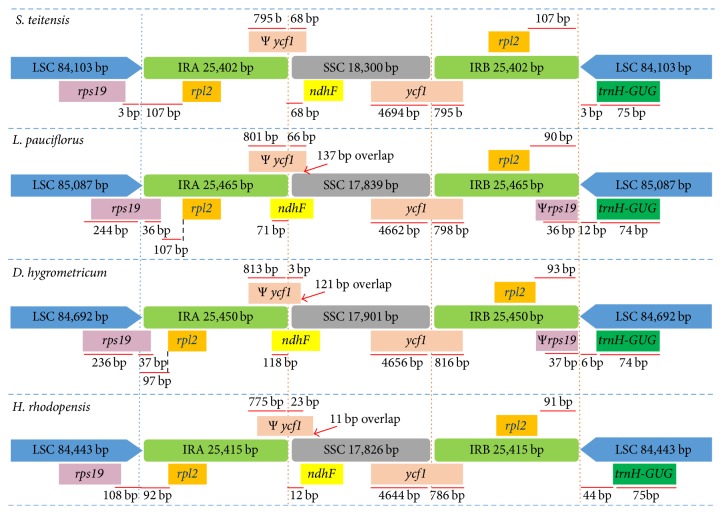
Comparison of LSC, SSC, and IR junctions among Gesneriaceae species. Ψ indicates a pseudogene. Genes above the regions are direct while genes below are reverse.

Comparing the LSC-IR-SSC junctions and their adjacent genes between* Streptocarpus teitensis*,* Lysionotus pauciflorus*,* Dorcoceras hygrometricum*, and* Haberlea rhodopensis* revealed some notable variations (Figure 4). The LSC-IRA junction had expanded into the gene* rps19* in two species (*Lysionotus pauciflorus*, 36 bp, and* Dorcoceras hygrometricum*, 37 bp) while in* Streptocarpus teitensis* and* Haberlea rhodopensis* the gene was 3 bp and 108 bp away from the junction, respectively. This resulted in the two species (*Lysionotus pauciflorus* and* Dorcoceras hygrometricum)* having a pseudogene of the* rps19* of 36 and 37 base pairs, respectively, at the IRB-LSC junction. A pseudogenized* ycf1* occurred at the IRA-SSC junctions in all species as a result of the extension of SSC-IRB junction into the* ycf1* gene, with variable extensions of the gene into the SSC region observed in the four species. An overlap of* Ψycf1* and* ndhF* genes was observed in* Dorcoceras hygrometricum* (121 bp),* Lysionotus pauciflorus* (137 bp), and* Haberlea rhodopensis* (11 bp) while in* Streptocarpus teitensis* the two genes joined each other. There was similarity in the SSC-IRB junction, in which* ycf1* gene occurred in all species, although the sizes varied with* Streptocarpus teitensis* having the largest (5,489 bp) and* Haberlea rhodopensis* the smallest (5,430 bp). A previous study on Rosaceae [27] observed the IRB-LSC junction to be characterized by the genes* rpl2* (IRB) and* trnH-GUG* (LSC). Similar observations were made in two Gesneriaceae species under the present study, while the species* Lysionotus pauciflorus* and* Dorcoceras hygrometricum* had a pseudogene of* rps19.* Additionally, the IRB-LSC junction occurred away from the gene* trnH-GUG* at variable lengths (3–44 base pairs) in the four species.

### 3.5. Phylogenetic Analysis

**Figure 5:**
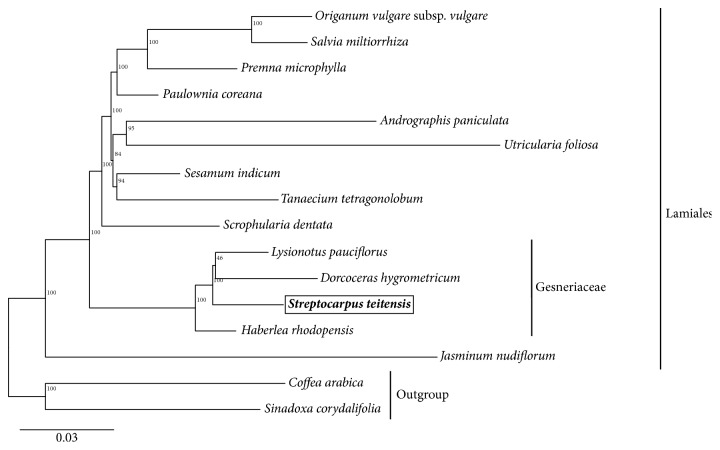
Maximum Likelihood phylogenetic tree of 14 species of Lamiales, and two other Asterid species as outgroups based on 67 chloroplast genome protein-coding genes' sequences.

The accessibility of numerous complete genomes sequenced has paved way for phylogenomics, a new method which has been observed to significantly offer solution to evolutionary issues by use of many characters [50, 51] contrary to the original phylogenetics. Previously, complete chloroplast genomes have helped resolve identity issues in the basal families of angiosperms [52]. In the Maximum Likelihood tree, 11 of the total 14 nodes had observed bootstrap values of ≥95%, with 10 of these having bootstrap values of 100%, while only three had low bootstrap values. The outcome displayed that* S. teitensis* clustered more closely with* Lysionotus pauciflorus* and* Dorcoceras hygrometricum* than with* Haberlea rhodopensis* (Figure 5). Additionally, at the order level, the family Gesneriaceae closely allied to family Scrophulariaceae. Generally, all the 14 species formed a lineage (Lamiales) noticeably distinct from the two out-group species.

## 4. Conclusion

Gesneriaceae is one of the important families in the order Lamiales as it is traded for its attractive flowers but their chloroplast genomes are understudied. Our research described the complete cp genome of* Streptocarpus teitensis*, a critically endangered species with one surviving population. This was the first whole cp genome to be reported in the genus* Streptocarpus* and the fourth in the family Gesneriaceae. The cp genome revealed genetic features and arrangement typical of the angiosperm cp genome. It also comprised 116 unique genes of which 80 are protein-coding, 32 tRNAs, and 4 rRNAs. Comparisons of the LSC/IR/SSC junctions in Gesneriaceae exposed some outstanding differences in the gene arrangements. Study of the phylogenetic tree revealed that* Lysionotus pauciflorus* and* Dorcoceras hygrometricum* were closer to* Streptocarpus teitensis* than* Haberlea rhodopensis*, while the family Scrophulariaceae was close to Gesneriaceae. This research informs the genetic structure of this endangered species and compares it to other members of the family and the order Lamiales.

## Figures and Tables

**Table 1:** The functional classification of genes found in *Streptocarpus teitensis* chloroplast genome.

Function	Group of genes	Gene names
Photosynthesis	Photosystem 1	*psaA, psaB, psaC, psaI, psaJ*
	Photosystem 11	*psbA, psbB, psbC, psbD, psbE, psbF, psbH, psbI, psbJ, psbK, psbL, psbM, psbN, psbT, psbZ*
	NADH dehydrogenase	*ndhA* ^*∗*^ *, ndhB* ^*∗*^ *(x2), ndhC, ndhD, ndhE, ndhF, ndhG, ndhH, ndhI, ndhJ, ndhK*
	ATP synthase	*atpA, atpB, atpE, atpF* ^*∗*^ *, atpH, atpI*
	Cytochrome b/f complex	*petA, petB, petD, petG, petL, petN*
	RubisCO large subunit	*rbcL*

Self-replication	RNA polymerase	*rpoA, rpoB, rpoC1* ^*∗*^ *, rpoC2*
	Ribosomal proteins (Large Sub-unit)	*rpl2* ^*∗*^ *, rpl14, rpl16, rpl20, rpl22, rpl23(x2), rpl32, rpl33, rpl36*
	Ribosomal proteins (small subunit)	*rps2, rps3, rps4, rps7(x2), rps8, rps11, rps12(x2), rps14, rps15, rps16* ^*∗*^ *, rps18, rps19*
	Ribosomal RNAs	*rrn4.5(x2), rrn5(x2), rrn16(x2), rrn23(x2)*
	Transfer RNAs	*trnA-UGC* ^*∗*^ * (x2), trnC-GCA, trnD-GUC, trnE-UUC, trnF-GAA, trnG-GCC* ^*∗*^ *, trnG-UCC, trnH-GUG*
		*trnI-CAU(x2), trnI-GAU* ^*∗*^ *(x2), trnK-UUU* ^*∗*^ *, trnL-CAA(x2), trnL-UAA* ^*∗*^ *, trnL-UAG, trnfM-CAU*
		*trnN-GUU(x2), trnP-UGG, trnQ-UUG, trnR-ACG(x2), trnR-UCU, trnS-GCU, trnS-GGA, trnS-UGA*
		*trnT-GGU, trnT-UGU, trnV-GAC(x2), trnV-UAC* ^*∗*^ *, trnW-CCA, trnY-GUA, trnP-GGG, trnM-CAU *

Proteins of unknown function		*ycf1(x2), ycf2(x2), ycf3* ^*∗∗*^ *, ycf4*

Other genes	Protease	*clpP* ^*∗∗*^
	Maturase	*matK*
	Translational initiation factor	*infA*
	Envelope membrane protein	*cemA*
	Subunit of acetyl-CoA-carboxylase	*accD*
	c-type cytochrome synthesis	*ccsA*

*∗* marks genes with one intron; *∗∗* marks genes with two introns; (*x2*) shows genes with duplicates.

**Table 2:** The genes with introns in the *Streptocarpus teitensis* chloroplast genome and the length of the exons and introns.

Gene	Region	Exon 1 (bp)	Intron 1 (bp)	Exon 2 (bp)	Intron 2 (bp)	Exon 3 (bp)
*atpF*	LSC	472	633	144		
*rps16*	LSC	207	1636	48		
*rpl2*	IR	435	670	393		
*rpoC1*	LSC	1620	784	456		
*ndhA*	SSC	540	1069	552		
*ndhB*	IR	756	679	777		
*trnA-UGC*	IR	38	834	35		
*trnI-GAU*	IR	35	935	42		
*trnK-UUU*	LSC	35	2493	37		
*trnG-GCC*	LSC	23	704	37		
*trnL-UAA*	LSC	37	469	50		
*trnV-UAC*	LSC	37	575	38		
*ycf3*	LSC	150	712	228	688	129
*ClpP*	LSC	234	631	297	819	69

**Table 3:** The AT and GC% in different regions of *Streptocarpus teitensis *cp genome.

Region	Length (bp)	A (%)	T (%)	G (%)	C (%)	GC (%)
LSC	84,103	31.57	32.88	17.36	18.18	35.54
SSC	18,300	34.13	34.5	15.15	16.22	31.37
IRA	25,402	28.46	28.34	22.43	20.77	43.2
IRB	25,402	28.34	28.45	20.77	22.43	43.2
Total genome	153,207	30.82	31.59	18.5	19.08	37.58

**Table 4:** Number of SSR repeats in *Streptocarpus teitensis *chloroplast genome.

Repeat sequences	Number of repeats											Total
	3	4	5	6	7	8	9	10	11	12	13	
A/T						55	33	8	5	4	1	106
C/G						4	2					6
AG/CT			2									2
AT/AT			4	1		1						6
AAC/GTT	10											10
AAG/CTT	20	1										21
AAT/ATT	18	1										19
AGC/GCT	6											6
AGG/CCT	4											4
ATC/GAT	5											5
AAAT/ATTT	3											3
AATC/GATT	2											2
AATT/AATT	1											1
AGAT/ATCT	2											2
AAAAG/CTTTT	2											2
AATTC/GAATT	1											1
Total												196

**Table 5:** Comparison of the features of four Gesneriaceae chloroplast genomes.

Feature	*Streptocarpus teitensis*	*Dorcoceras hygrometricum*	*Lysionotus pauciflorus*	*Haberlea rhodopensis*
Genome size (bp)	153,207	153,493	153,856	153,099
Large single copy (bp)	84,103	84,692	85,087	84,443
Small single copy (bp)	18,300	17,901	17,839	17,826
Inverted repeats (bp)	25,402	25,450	25,465	25,415
GC content in LSC (%)	35.5	35.6	35.4	35.7
GC content in SSC (%)	31.4	36.4	36.6	31.7
GC content in IR (%)	43.2	40.7	40.6	43.3
Overall AT content (%)	62.4	62.4	62.5	62.2
Overall GC content (%)	37.6	37.6	37.5	37.8

**Table 6:** Comparison of the features of *Streptocarpus teitensis *with other 11 Lamiales chloroplast genomes.

Species	Family	LSC (bp)	SSC (bp)	IR (bp)	Total (bp)	CG content%	Accession number
*Streptocarpus teitensis*	Gesneriaceae	84,103	18,300	25,402	153,207	37.58	MF596485
*Cistanche deserticola*	Orobanchaceae	49,130	8,819	22,354	102,657	36.8	KC128846
*Sesamum indicum*	Pedaliaceae	85,170	17,872	25,141	153,324	38	JN637766
*Premna microphylla*	Lamiaceae	86,078	17,689	25,763	155,293	37.9	KM981744
*Salvia miltiorrhiza*	Lamiaceae	82,695	17,555	25,539	151,328	38	JX312195
*Origanum vulgare *subsp.* vulgare*	Lamiaceae	83,136	17,745	25,527	151,935	38	JX880022
*Utricularia foliosa*	Lentibulariaceae	82,720	17,481	25,325	150,851	37.32	KY025562
*Andrographis paniculata*	Acanthaceae	82,459	17,190	25,300	150,249	38.3	NC022451
*Tanaecium tetragonolobum*	Bignoniaceae	84,612	17,586	25,789	153,776	38.3	KR534325
*Paulownia coreana*	Paulowniaceae	85,241	17,736	25,784	154,545	38	KP718622
*Scrophularia dentata*	Scrophulariaceae	84,058	17,449	25,523	152,553	38	KT428154
*Jasminum nudiflorum*	Oleaceae	92,877	13,272	29,486	165,121	38	NC_008407
